# Supplementary material for: OliTag-seq enhances in cellulo detection of CRISPR-Cas9 off-targets
Source: Commun Biol. 2024 Jun 6;7:696. doi: 10.1038/s42003-024-06360-w (PMC11156888; doi:10.1038/s42003-024-06360-w)
Supplement: Supplementary file 2 — Supplementary Information [file 42003_2024_6360_MOESM2_ESM.pdf]

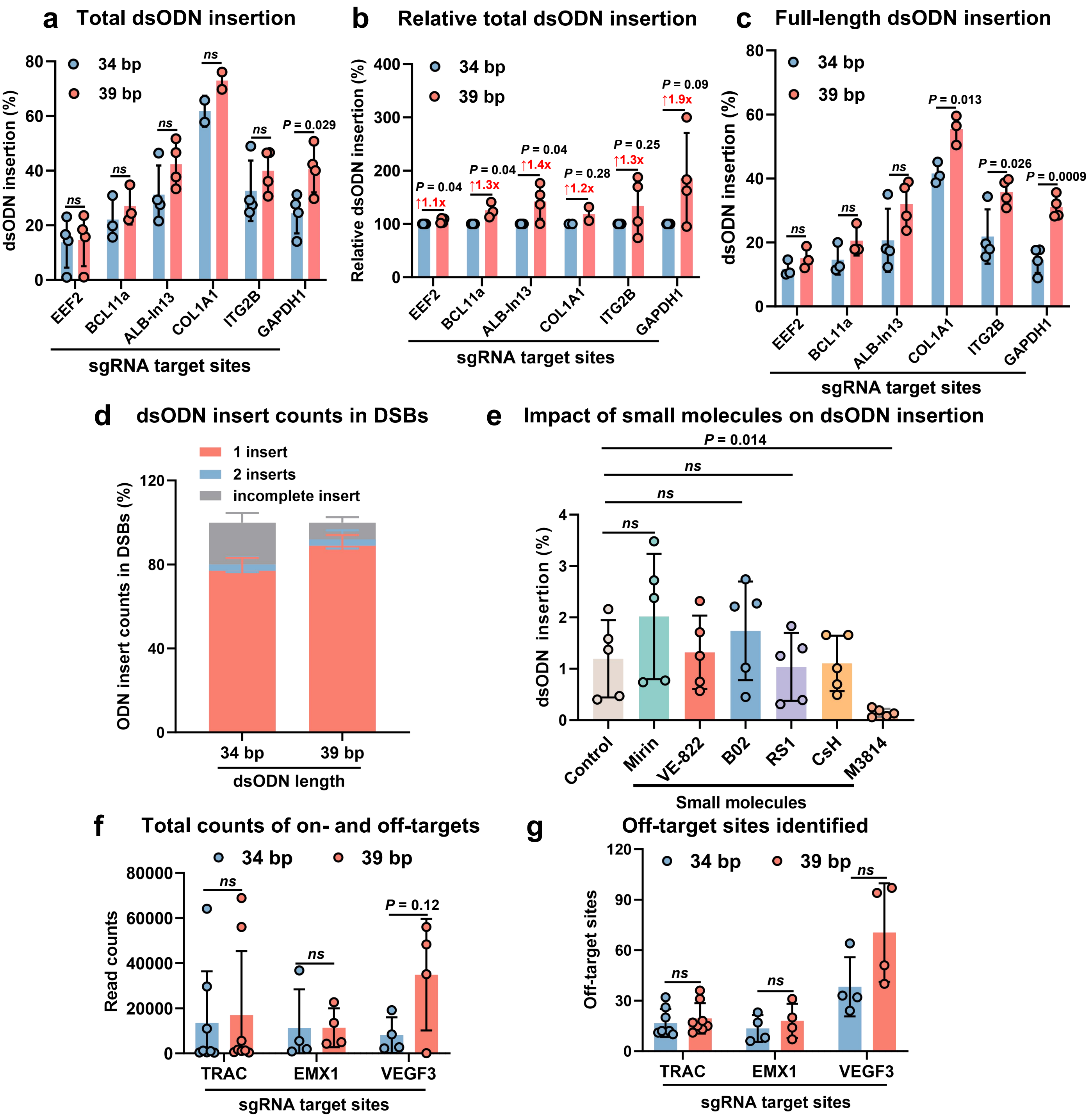


**Supplementary Fig. 1: Comparative Efficiency of 39 bp vs. 34 bp dsODN in Targeted Integration. a** dsODN Insertion Efficiency Across Targets: Displays a comparison of total dsODN insertion efficiencies between 34 bp and 39 bp ODNs across six clinical targets (n = 3-4 per site), calculated by the ratio of ODN insert counts to total editing event reads at each site. **b** Relative dsODN Integration Frequencies: Presents integration frequencies of dsODNs as relative values, normalizing the insertion efficiency of 39 bp ODN to that of 34 bp. **c** Full-Length dsODN Insertion Comparison: Compares the insertion of full-length dsODN between 34 bp and 39 bp variants at the six target sites (n = 3-4 per site), efficiency determined by the ratio of full-length insert counts to total editing event reads. **d** ODN Insertion in DSB Events: Evaluates the insertion frequency of 34 bp and 39 bp ODNs at Cas9-induced DSBs, considering three types of integration events. **e** NHEJ Pathway Influence on dsODN Integration: Analyzes the impact of various small molecules on the frequency of NHEJ-mediated dsODN integration (n = 5), with M3814, an NHEJ pathway inhibitor, as a control. **f** Sequencing Reads Comparison for DSB Tagging: Contrasts the sequencing read counts for DSBs tagged with 34 bp versus 39 bp ODNs. **g** Off-Target Site Identification: Compares off-target sites mapped in *TRAC*, *EMX1*, and *VEGF3* (n = 4-6 per site) using both ODN sizes. Data are represented as mean ± s.d., with *P* values determined by a paired two-tailed Student's t-test. Adjusted P values and fold changes are provided for enhanced interpretation of the significance.

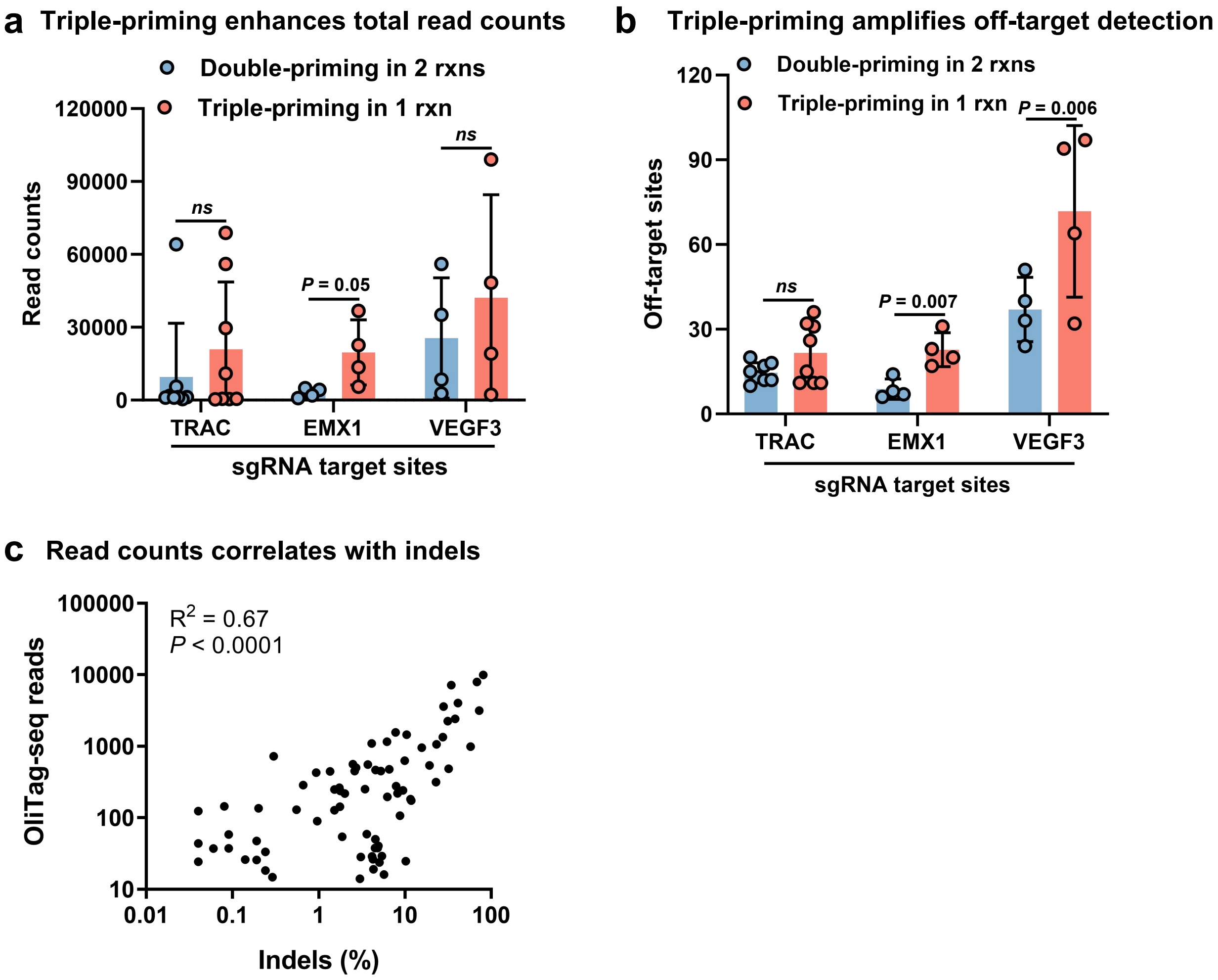


**Supplementary Fig. 2: Enhanced Detection of Off-Targets in OliTag-seq Using Triple Priming. a** Read Count Amplification with Triple Priming: Demonstrates that triple priming within a single reaction boosts the read counts for both on-target and off-target sequences compared to double priming across two reactions, standardizing for equal amounts of genomic DNA and sequencing depth (n = 4 or 7 per site). **b** Off-Target Event Identification: Shows that a single-reaction triple-priming method identifies a greater number of off-target events than the traditional double-priming technique (n = 4 or 7 per site). **c** Editing Frequency and Read Count Correlation: Presents the correlation between total editing frequencies and the corresponding sequencing read counts.Data across panels are presented as mean ± s.d., with *P* values determined by a paired two-tailed Student's t-test. Adjusted *P* values are provided alongside the fold changes where applicable, with "ns" indicating a lack of statistical significance ($P > 0.05$).

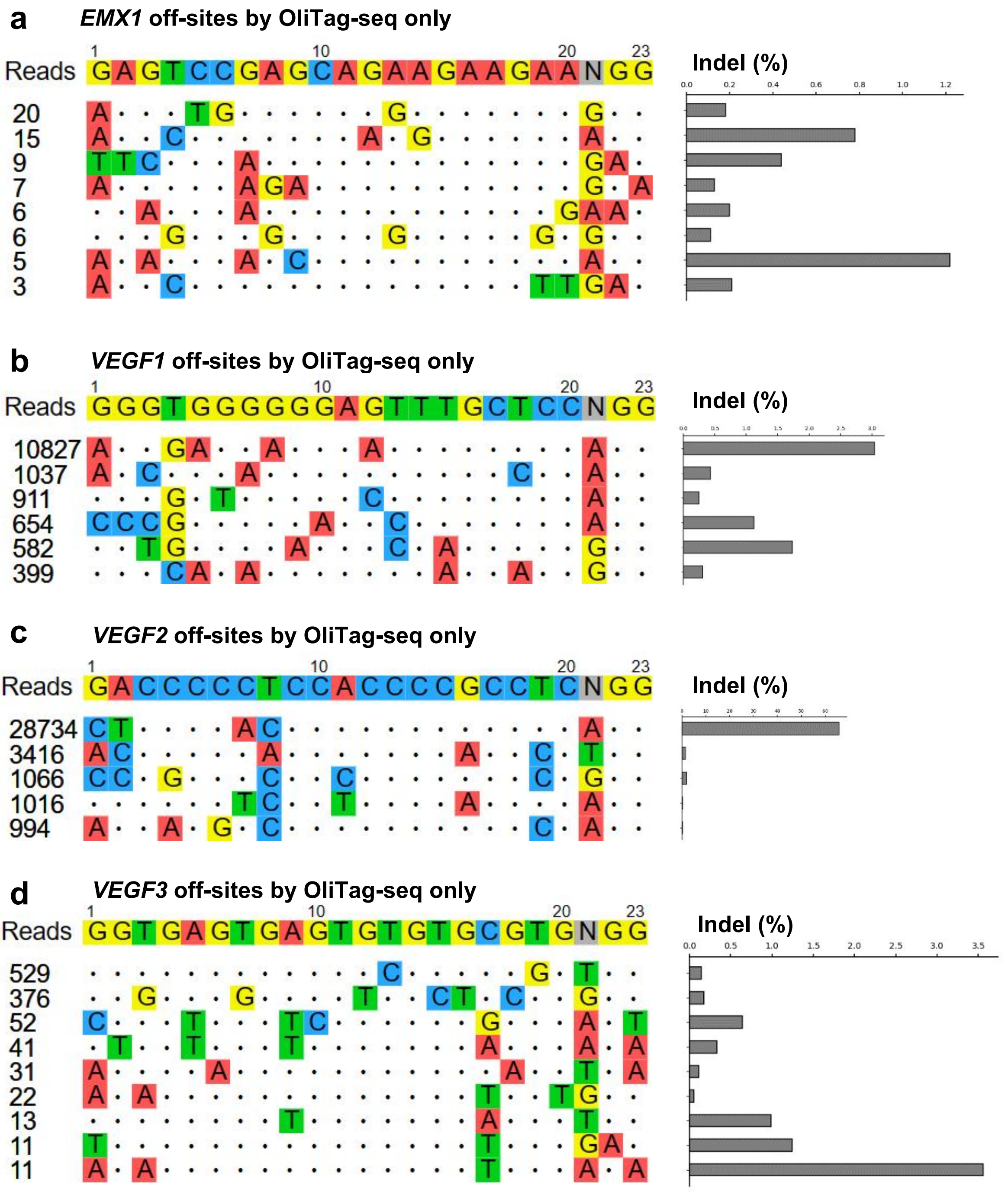


**Supplementary Fig. 3: Rigorous Validation of Novel Off-Target Sites in U2OS Cells by OliTag-seq. a-d** Indel Frequency Analysis at Newly Identified Off-Target Sites: This figure segment scrutinizes indel frequencies at newly revealed off-target locations identified by OliTag-seq but not by GUIDE-seq in U2OS cells targeted with sgRNAs for *EMX1* (**a**), *VEGF1* (**b**), *VEGF2* (**c**), and *VEGF3* (**d**). These analyses were performed on cells edited with the sgRNAs without the integration of the ODN template, with detection via Illumina sequencing. The data underscore the enhanced detection capabilities of OliTag-seq in identifying authentic novel off-target sites, as corroborated by subsequent independent editing assays, thus confirming the robustness and heightened sensitivity of OliTag-seq over GUIDE-seq for off-target discovery.

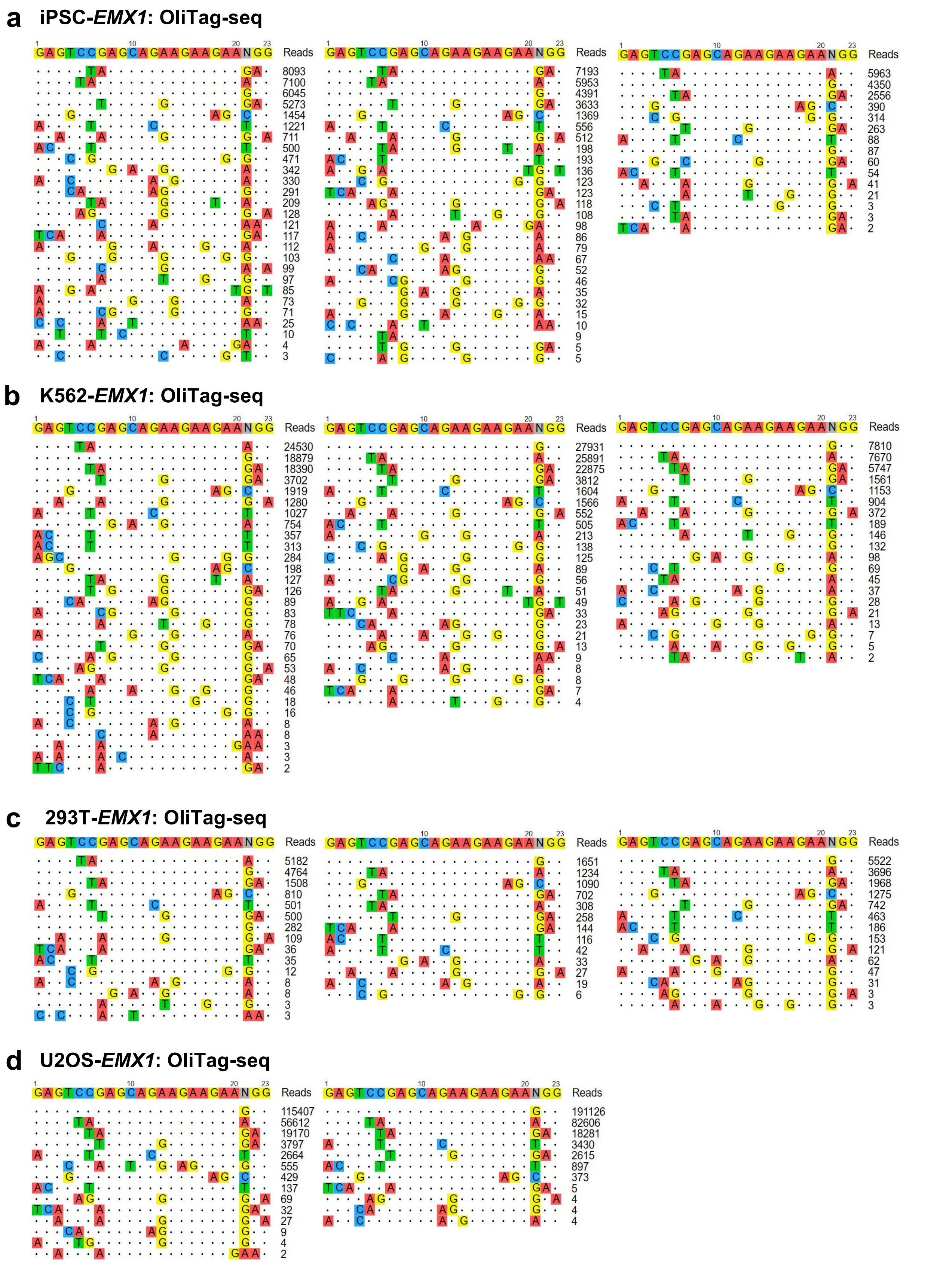


**Supplementary Fig. 4: Cross-Cell Line Analysis of EMX1 Off-Target Sites by OliTag-seq. a-d** Cross-Sectional Off-Target Visualization: Presents a comparative visualization of off-target sites for sgRNA targeting the *EMX1* gene locus across four different cell lines: iPSC (**a**), K562 (**b**), 293T (**c**), and U2OS (**d**). For each cell line, the analysis includes multiple biological replicates (n = 2 or 3). The on-target sequence with its protospacer adjacent motif (PAM) is highlighted at the top for reference. Below, the mismatched nucleotides at each off-target site are indicated with color coding for clarity. Accompanying OliTag-seq read counts for each off-target are provided to the right, offering quantitative insights. The figure also details the consistency of off-target site detection across biological replicates, underscoring the reliability and reproducibility of the OliTag-seq method.

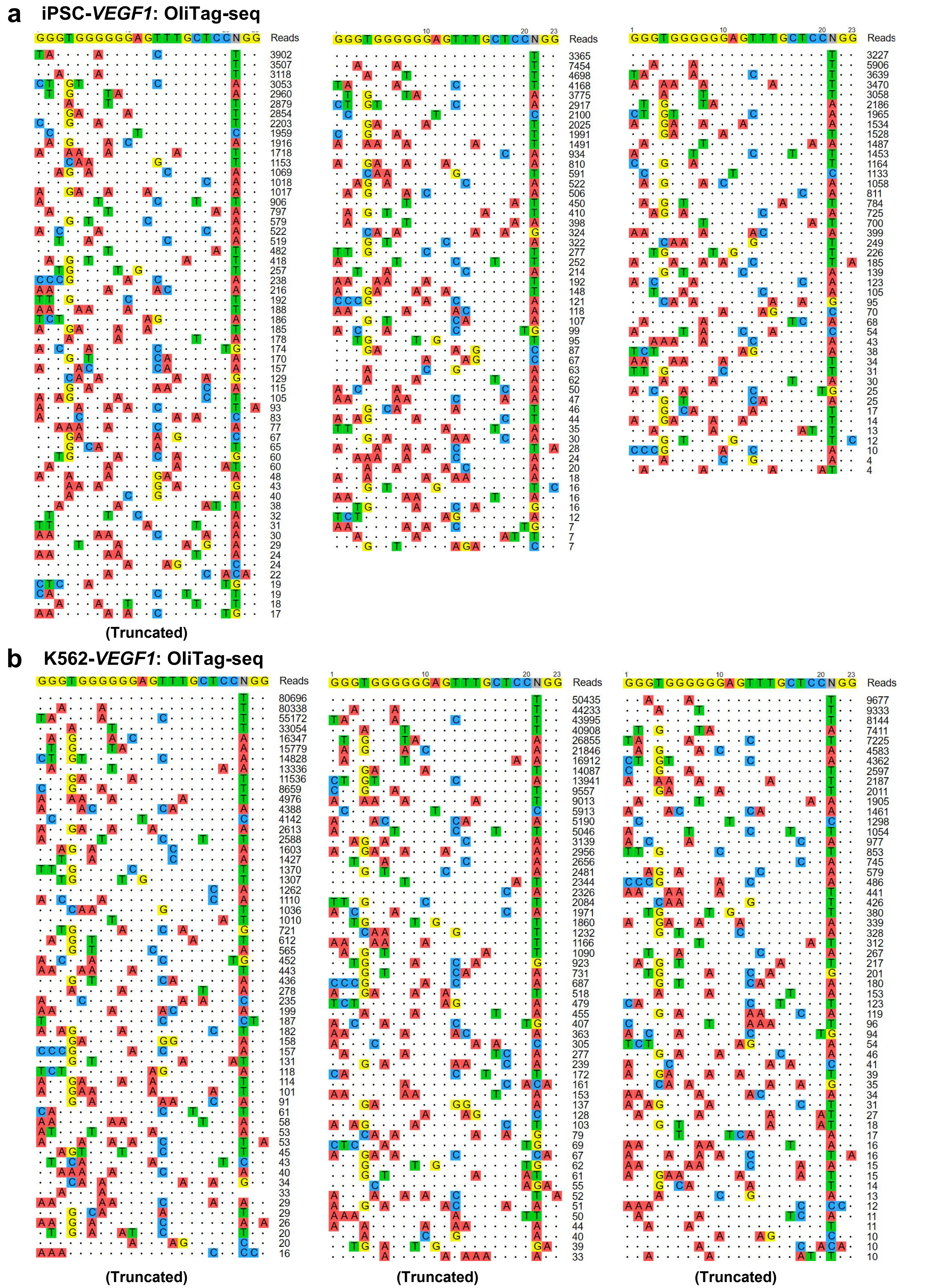


**Supplementary Fig. 5: OliTag-seq Mapping of VEGF1 Off-Targets in iPSC and K562 cells. a, b** Detailed Off-Target Site Mapping: This figure displays the localization of off-target sites identified by OliTag-seq for an sgRNA targeting the *VEGFA site 1* within two cell types: iPSC (**a**) and K562 (**b**). For each cell line, off-targets were analyzed across three biological replicates. The precise on-target sequence, complete with the protospacer adjacent motif (PAM), is prominently exhibited at the top of each panel. Deviations from the expected sequence at off-target locations are marked in color to emphasize the mismatches. Adjacent to each off-target site, OliTag-seq provides quantified read counts, reflecting the detection frequency and enabling a direct comparison of off-target editing events between the two cellular environments.

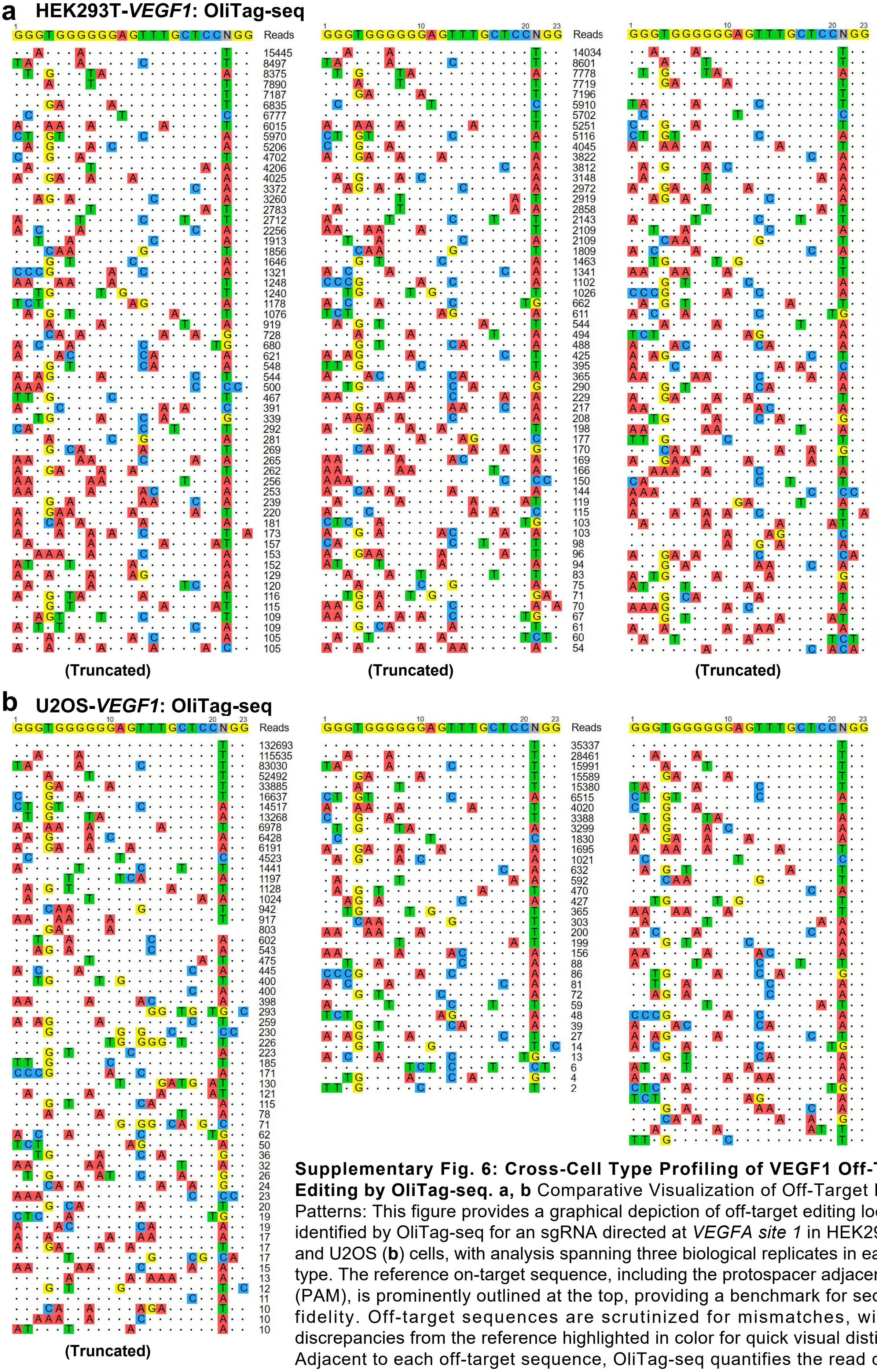


**Supplementary Fig. 6: Cross-Cell Type Profiling of VEGF1 Off-Target Editing by OliTag-seq. a, b** Comparative Visualization of Off-Target Editing Patterns: This figure provides a graphical depiction of off-target editing locations identified by OliTag-seq for an sgRNA directed at *VEGFA site 1* in HEK293T (**a**) and U2OS (**b**) cells, with analysis spanning three biological replicates in each cell type. The reference on-target sequence, including the protospacer adjacent motif (PAM), is prominently outlined at the top, providing a benchmark for sequence fidelity. Off-target sequences are scrutinized for mismatches, with any discrepancies from the reference highlighted in color for quick visual distinction. Adjacent to each off-target sequence, OliTag-seq quantifies the read counts, offering a metric of occurrence frequency for each identified editing event. This side-by-side comparison across two cell lines showcases the versatility and sensitivity of OliTag-seq in detecting a spectrum of CRISPR-Cas9-induced off-target effects.

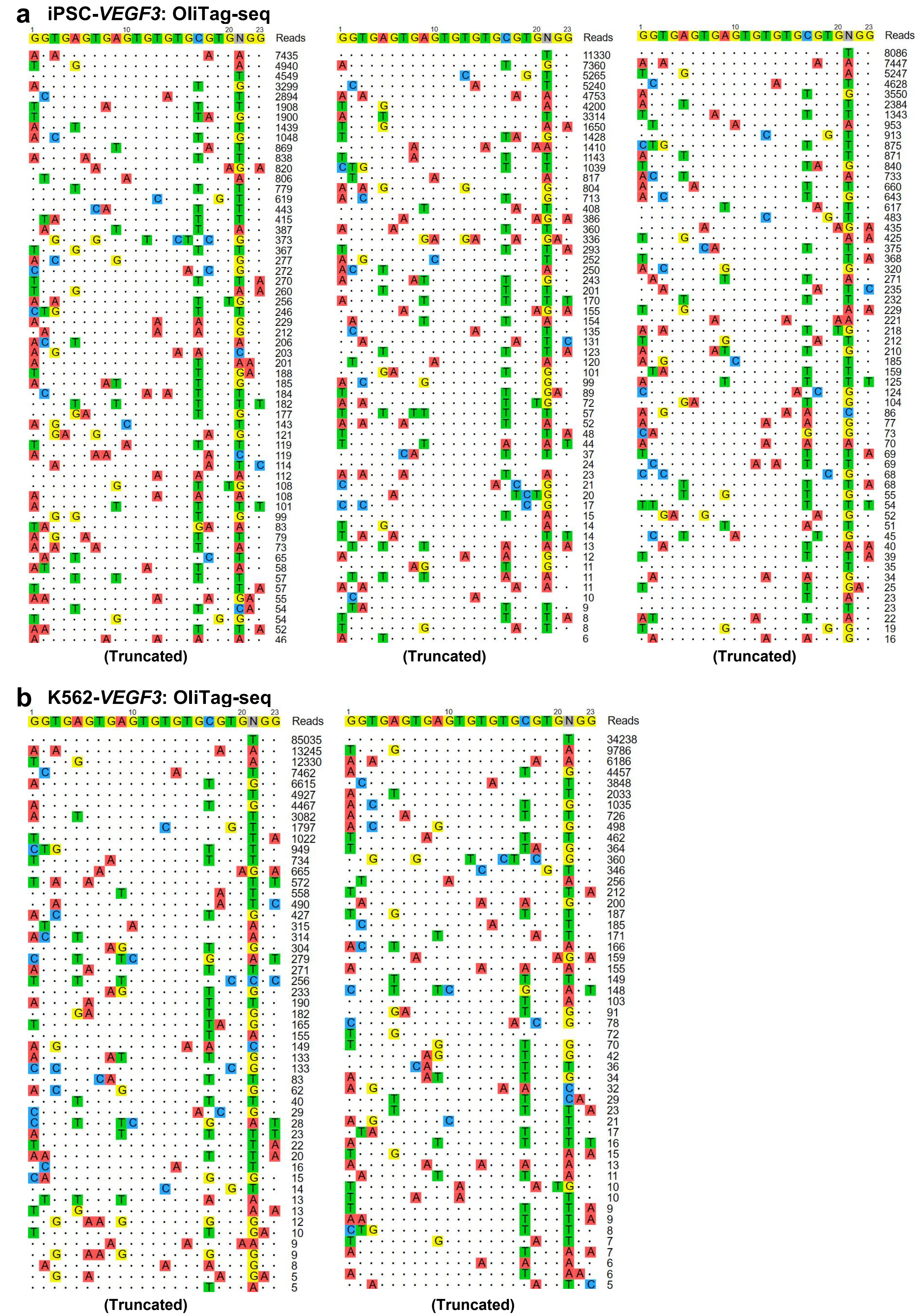


**Supplementary Fig. 7: *VEGF3* off-target sites detected by OliTag-seq in iPSC and K562 cells. a, b** Visualization of off-target sites across biological replicates (n = 2 or 3) for an sgRNA targeting *VEGFA site 3* in iPSCs (**a**), K562 cells (**b**). The on-target sequence with PAM is displayed on the top line. Mismatches in off-target sites are highlighted in color. OliTag-seq read counts are presented on the right.

**a**

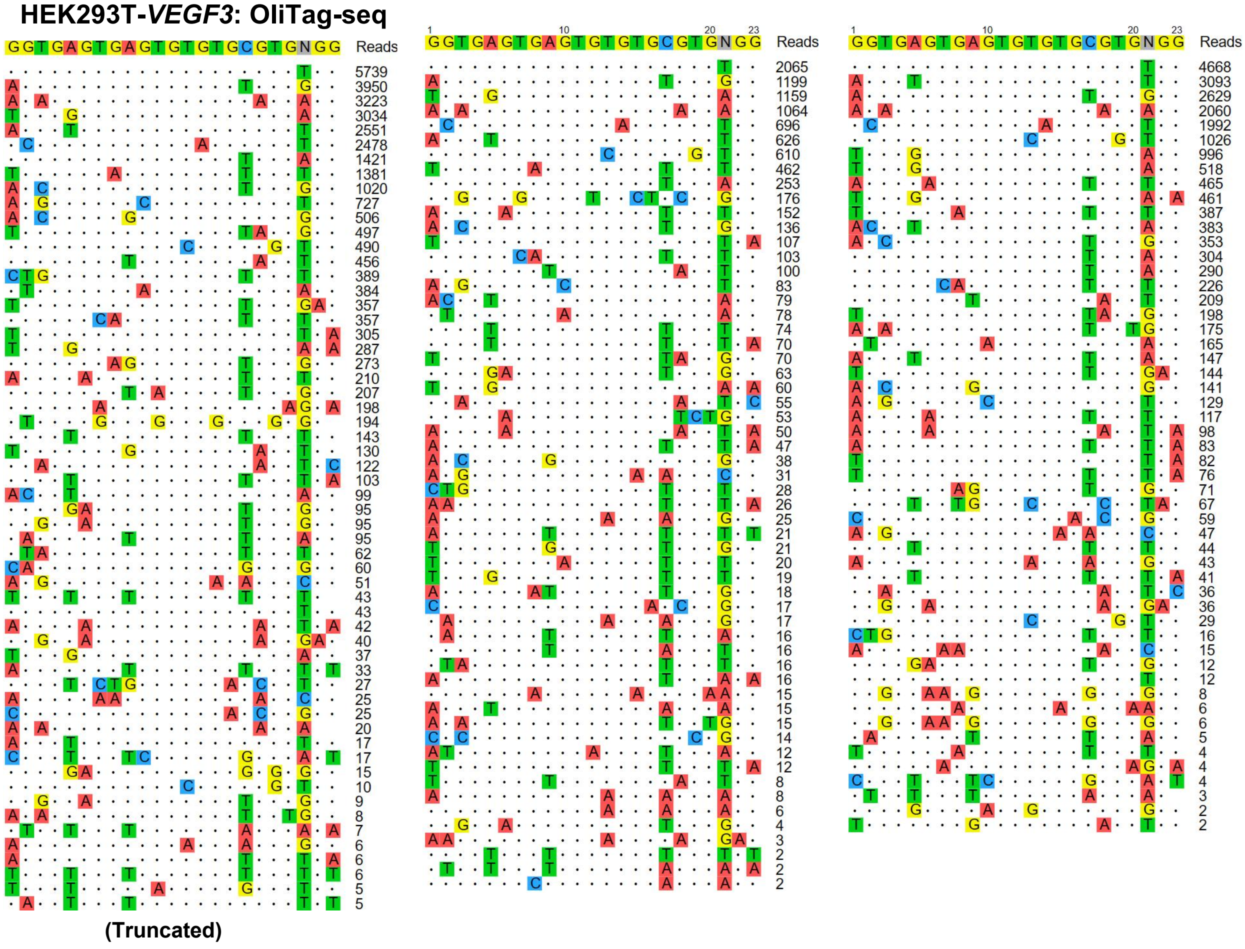


**b**

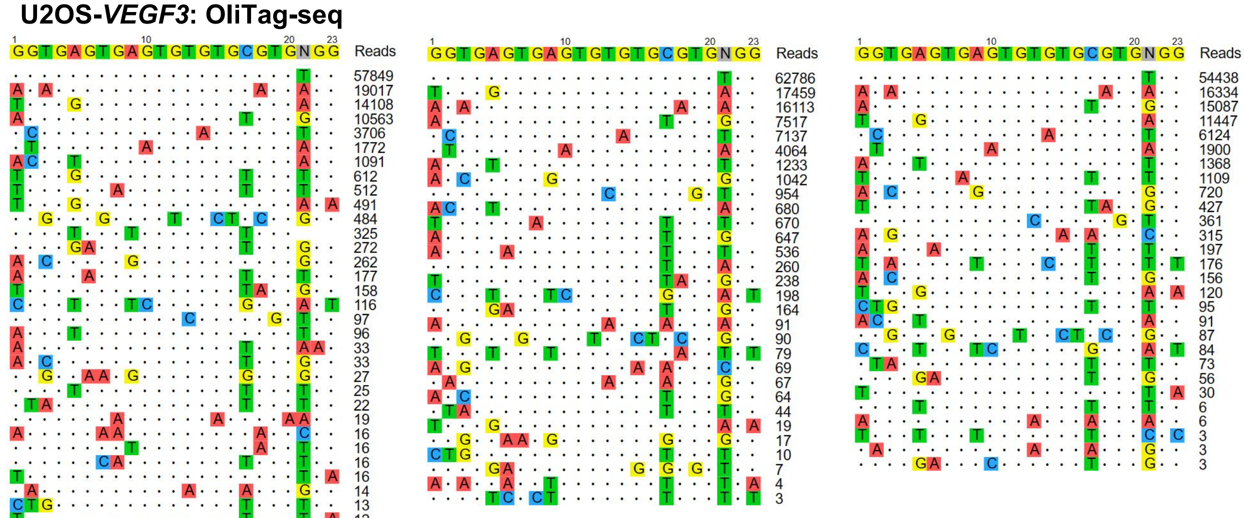


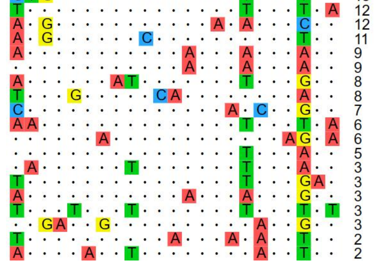

**Supplementary Fig. 8: OliTag-seq Surveillance of VEGF3 Off-Target Activity in Diverse Cell Lines. a, b** Detailed Representation of Off-Target Editing: Showcases the precision of OliTag-seq in detecting off-target sites for an sgRNA directed at *VEGFA site 3* within HEK293T (**a**) and U2OS (**b**) cells. The analysis includes results from three biological replicates per cell line, ensuring data reliability. The on-target sequence and its PAM are indicated at the top of each cell line's panel. Discrepancies from the on-target sequence are colored at each off-target site, allowing for an intuitive assessment of mismatch occurrences. To the right, the read counts from OliTag-seq are listed. This visualization affirms the efficacy of OliTag-seq in off-target detection and provides insights into the cell line-specific landscape of CRISPR-induced off-target mutations.

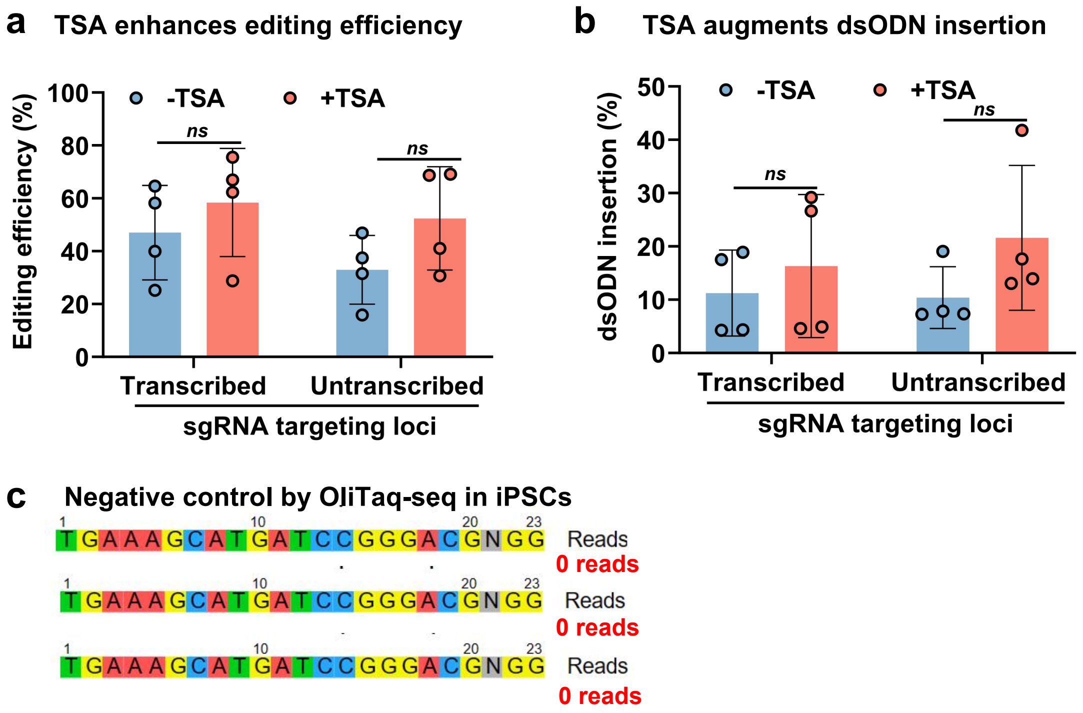


**Supplementary Fig. 9: The Potentiating Effect of TSA on CRISPR-Cas9 Off-Target Editing. a** TSA's Impact on Gene Editing Efficiency: Examines the influence of trichostatin A (TSA), a histone deacetylase inhibitor, on the total editing efficiency at both active (*AAVS1*, *CD326*, *EEF1A1*, *EEF2*) and inactive (*CCR5*, *CIITA*, *TRAC*, *HBG*) gene sites (n = 4), with site activity informed by TPM values from RNA-seq data. **b** TSA's Role in dsODN Integration: Assesses the effect of TSA treatment on the total dsODN insertion frequency at those mentioned above transcribed and untranscribed gene sites (n = 4). **c** Specific sgRNA Editing in the Presence of TSA: Presents data from iPSC-Cas9 cells treated with TSA (0.1 μM) targeting the sgRNA sequence TGAAAGCATGATCCGGGACG across three biological replicates, in the absence of sgRNA, to evaluate the specificity of TSA's effect on genomic editing. The results across these experiments are presented as mean ± standard deviation. Statistical significance is calculated using a paired two-tailed Student's t-test. Instances where differences are not statistically significant are annotated with "ns" ($P > 0.05$). This figure aims to elucidate TSA's ability to modulate the CRISPR-Cas9 editing landscape, potentially enhancing off-target event detection.

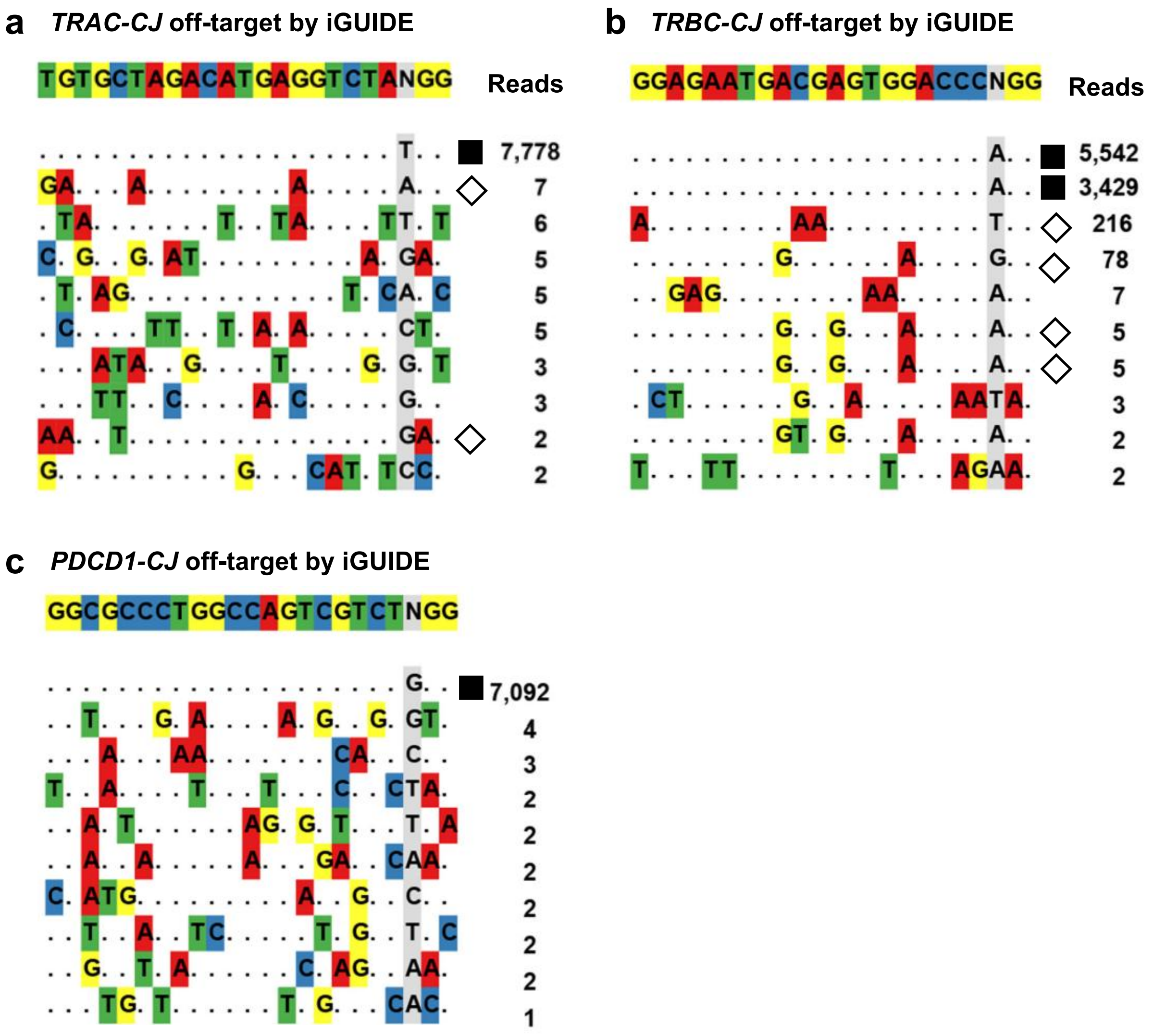


**Supplementary Fig. 10: Comparative Analysis of iGUIDE and OliTag-seq in Detecting Off-Target Sites.** **a-c**The figure provides a graphical representation of off-target sites for sgRNAs targeting three loci: *TRAC-CJ* (**a**), *TRBC-CJ* (**b**), and *PDCD1-CJ* (**c**). The off-target data visualized in the figure is sourced from iGUIDE-seq, as reported in the study by E. A. Stadtmauer et al., 2020. For each locus, mismatches from the expected genomic sequence at identified off-target sites are indicated with color coding for easy visualization. The primary on-target site for each sgRNA is denoted with a black square for reference. Additionally, off-target sites detected by OliTag-seq are marked with an open diamond, facilitating a direct comparison between the two methods regarding off-target identification capabilities.

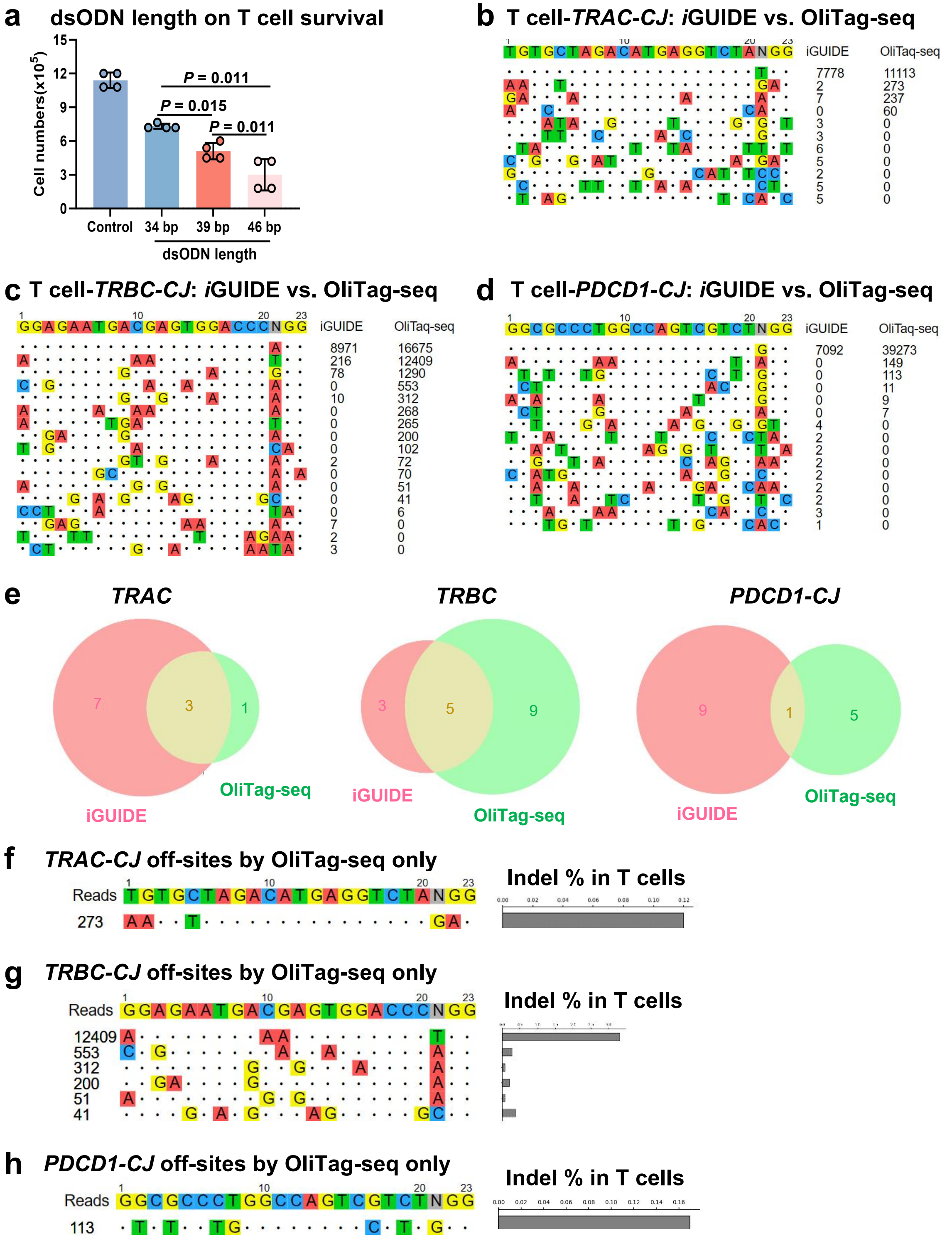


**Supplementary Fig. 11: Comparative Analysis of Off-Target Detection in CAR-T Related Loci by OliTag-seq and iGUIDE. a** dsODN Length Effects in T Cells: Analyzes the impact of varying dsODN lengths on T cell survival. A consistent number of T cells were electroporated for each experimental setup with 50 pmol of dsODN. Results are quantified and expressed as mean ± s.d., with statistical significance determined by a paired two-tailed Student's t-test. **b-d** Off-Target Detection in T Cells: Presents data for sgRNAs targeting CAR-T related loci: *TRAC-CJ* (**b**), *TRBC-CJ* (**c**), and *PDCD1-CJ*(**d**). These experiments were performed on primary T cells complexed with annealed crRNA:tracrRNA. Off-target mismatches are visually differentiated with color highlights. To the right, read counts from iGUIDE-seq and OliTag-seq are listed, providing a quantitative comparison. **e** Overlap of Detected Off-Targets by iGUIDE and OliTag-seq: Venn diagrams showcase the concordance between off-target sites identified by iGUIDE and those verified by OliTag-seq for the sgRNAs targeting *TRAC-CJ*, *TRBC-CJ*, and *PDCD1-CJ*. **f-h** Indel Frequency Validation at Off-Target Sites: Reports the frequencies of insertions and deletions (indels) as detected by Illumina amplicon sequencing at off-target sites for sgRNAs targeting *TRAC-CJ* (**f**), *TRBC-CJ* (**g**), and *PDCD1-CJ* (**h**). These indel frequencies were assessed in gene-edited primary T cells without the dsODN template to validate off-target occurrences. This figure underscores the precision of OliTag-seq in characterizing off-target effects in the context of CAR-T cell therapies, highlighting the technology's potential for detecting unintended edits with higher sensitivity compared to previous methods.

| Target site | sgRNA sequence |
|---|---|
| sgEMX1 | GAGTCCGAGCAGAAGAAGAA |
| sgVEGF1 | GGGTGGGGGGAGTTTGCTCC |
| sgVEGF2 | GACCCCCTCCACCCCGCCTC |
| sgVEGF3 | GGTGAGTGAGTGTGTGCGTG |
| sgAAVS1b | GGGGCCACTAGGGACAGGAT |
| sgCD326a | GTTCGGGCTTCTGCTTGCCG |
| sgEEF1A1-In1a | GTAGTCATCCTTACCCAA |
| sgEEF2 | gTTCCTGGACAAATTGTAGG |
| sgCCR5 | GCTGTGTTTGCGTCTCTCCC |
| sgCIITA | gAAATCTCTGAGGCTGGAAC |
| sgTRAC | gTGGGGAAGAAGGTGTCTTC |
| sgHBG2 | gCCTTGAAAGCTCTGCATCA |
| sgTRAC-CJ | gTGTGCTAGACATGAGGTCTA |
| sgTRAC-MH | gTCAGGGTTCTGGATATCTGT |
| sgTRBC-CJ | GGAGAATGACGAGTGGACCC |
| sgTRBC-MH | gAGAGATCTCCCACACCCAAA |
| sgPDCD1-CJ | GGCGCCCTGGCCAGTCGTCT |

**Table S1: List of target sites and sgRNA sequence.**
