# Supplementary material for: OliTag-seq enhances in cellulo detection of CRISPR-Cas9 off-targets
Source: Commun Biol. 2024 Jun 6;7:696. doi: 10.1038/s42003-024-06360-w (PMC11156888; doi:10.1038/s42003-024-06360-w)
Supplement: Supplementary file 3 — Description of Additional Supplementary Files [file 42003_2024_6360_MOESM3_ESM.pdf]

Description of Additional Supplementary Files

File name: Supplementary Data 1
Description: The source data behind the Figure 4 A-D graphs in the paper.

File name: Supplementary Data 2
Description: The source data behind the Figure 5 D-F graphs in the paper.

File name: Supplementary Data 3
Description: The source data behind the Figure 6 A-B graphs in the paper.

File name: Supplementary Data 4
Description: The source data behind the Figure 7 graphs in the paper.

File name: Supplementary Data 5
Description: The name of the primer and its corresponding sequence used in our research.

File name: Supplementary Data 6
Description: The source data behind the Figure 3 graphs in the paper.
